# Surface Modification of Ti-35Nb-10Ta-1.5Fe by the Double Acid-Etching Process

**DOI:** 10.3390/ma11040494

**Published:** 2018-03-26

**Authors:** Joan Lario, Angélica Amigó, Francisco Segovia, Vicente Amigó

**Affiliations:** Instituto de Tecnología de Materiales, Universitat Politècnica de València, 46022 Valencia, Spain; anamma@upvnet.upv.es (A.A.); fsegovia@mcm.upv.es (F.S.); vamigo@mcm.upv.es (V.A.)

**Keywords:** titanium alloys, acid etching, surface roughness, topography, Ti-Nb-Ta-Fe, beta alloy

## Abstract

Surface topography and composition influence the osteoblastic proliferation and osseointegration rates, which favor the biomechanical stability of bone anchoring and implants. In recent years, beta titanium alloys have been developed, and are composed of biocompatible elements, have low elastic modulus, high corrosion resistance, and mechanical properties to improve the long performance behavior of biomaterials. In the present research, the influence of the acid-etching process was studied in Ti6Al4V ELI and Ti35Nb10Ta1.5Fe. Samples were etched in a two-step acid treatment. Surface roughness parameters were quantified under a confocal microscope, topography was studied by scanning electron microscopy, and surface composition was analyzed with energy dispersive X-ray spectroscopy. The results revealed that the two-step acid treatment changes the topography of the β alloy, increases the surface area, and changes the chemical composition of the surface. Two differentiated regions were identified in the Ti35Nb10Ta1.5Fe alloy after the acid-etching process: The α + β region with higher values of mean roughness due to the lower chemical resistance of this region; and the β region with lower values of roughness parameters.

## 1. Introduction

The development of new beta titanium alloys and surface treatments to improve the long performance of implants and to cut patient hospitalization times, recovery periods, and clinical implant revisions has attracted the interest of researchers [1]. The average lifetime of an orthopedic implant is 10–15 years, which means that patients must undergo difficult substitution surgeries with long rehabilitation periods [2]. Surface treatments that modify a material’s composition and surface topography may improve osseointegration rates and decrease implant failure probability [3]. 

Higher concentrations of vanadium particles on the tissue closest to the implant material have been mentioned in some titanium alloy studies. These particles are toxic, may have a carcinogenic effect for patients, and can cause interferences with cell growth [4,5,6,7]. At the beginning of the 1990s, the development of β titanium alloys commenced, mainly due to their higher fatigue and corrosion resistance levels and lower Young’s modulus. Those beta titanium alloys present less-toxic alloying elements (e.g., niobium, tantalum) which decrease Young’s modulus compared with commercially pure titanium and Ti-6Al-4V ELI alloys employed as biomaterials. The Young’s modulus mismatch between the implant and bone reduce the load transfer, increasing the bone reabsorption rate [8,9]. A metallic implant has to be biocompatible, which means that it does not cause any adverse response in its host. Implants must present the ability to perform a specific function in the body, such as promote protein and cell adhesion, cell proliferation, and favor apatite formation; they must also present elevated corrosion resistance, because they come into contact with body fluids [3]. Corrosion and wear processes release metal ions and particles that interfere with cell growth. β stabilization elements such as Nb, Ta, and Zr present elevated biocompatibility and corrosion resistance, which make them suitable for use as biomaterials [10]. 

The efforts made in recent years have focused on developing new β alloys composed of biocompatible elements, as well as new surface treatments to improve osseointegration rates. Powder metallurgy (P/M) is a manufacturing technology in which the microstructure is achieved through solid-state diffusion that enables titanium alloys with non-soluble phases to be obtained, which are impossible to obtain through melting and casting. These alloy types usually contain refractory elements with a high melting point and density. Depending on the selected manufacturing parameters, alloys may present a lack of diffusion, which ends in elevated microstructure heterogeneity [11]. The addition of Fe diminishes the α phase due to its greater beta stabilizer property [12]. Chemical compositions or charges on the surface of titanium implants differ according to their bulk composition and surface treatments. These parameters are critical for protein adsorption and cell attachment [13]. Nb and Ta have been used as safe alloying elements in Ti alloys because they are considered non-toxic, non-allergenic, and excellent β stabilizer elements [14]. 

The thickness of TiO_2_ passive film governs the metal ion release from a titanium implant to the human body, which determines titanium surface stability and biocompatibility. Modifying the roughness and surface area of implants can enhance protein absorption and cell proliferation, increase bone regeneration, and improve the short- and mid-term healing periods [15,16]. Surface topography can be modified by plasma-sprayed coatings, grit blasting, acid etching, and electrochemical processes, or by a combination of some of these [17]. Plasma-sprayed coatings present the problems of a heterogeneous layer degradation, mechanical failure, and fragmentation, and can also change the titanium alloy microstructure due to high coating temperatures [17,18]. Contamination particles such as aluminum oxide or silicon oxide from blasting affect surface composition and energy, and induce adverse cellular responses [19]. Acid-etching treatments are used to obtain a microroughness topography on the implant surface [20,21,22,23,24,25]. The dimensions of the achieved microvoids range from 0.4 to 4 µm [26,27,28,29]. The acid-etching process has been selected because it improves the osseointegration rate and biocompatibility compared with plasma-sprayed or smooth-machined surfaces [26,27,28,29,30,31,32].

The aim of this work was to study the effect of the double acid-etching process on the roughness and topography of the surface of Ti35Nb10Ta1.5Fe alloys obtained by conventional P/M, and to compare the results obtained with Ti6Al4V ELI from casting with the same surface treatment.

## 2. Materials and Methods

New beta titanium alloy with nominal composition Ti35Nb10Ta1.5Fe was obtained through PM process using blending elemental powder, provided by Atlantic Equipment Engineers (Upper Saddle River, NJ, USA): Ti 99.7% purity and <48 µm particle size, Nb 99.8% purity and <20 µm particle size, Ta 99.8% purity and <8 µm particle size; Fe 99.9% purity and <34 µm particle size by Höganäs. Blend powders were pressed at 600 MPa and sintered in a vacuum sintering furnace (<10^−4^ mbars). Samples were heated to 800 °C at 15 °C/min, held at that temperature for 30 min, and finally heated to 1250 °C at 10 °C/min, and then held for 180 min and cooled at 10 °C/min. Cylindrical floating dies, 20 mm diameter, were used to obtain round samples of 6 mm in thickness. The Ti6Al4V ELI bar was supplied by Carpenter Technology Corporation (Philadelphia, PA, USA) and machined on round samples with a diameter of 12.7 mm and a thickness of 4 mm. The density of the sintered samples was determined by the Archimedes method, in accordance with ASTM standard C373-88. Tensile specimens were tested by a Shimadzu AG-X plus mechanical tester (Shimadzu, Kyoto, Japan) with a crosshead speed of 0.5 mm/min, following standard ISO 3325. The ultrasonic technique was selected to measure Young’s modulus, for which the Karl Deutsch Echograph 1090 equipment was employed (Karl Deutsch, Wuppertal, Germany). 

The samples were wet-ground with 220, 500, 1000, and 1500 grit silicon carbide (SiC) paper before double acid-etching attack. In order to modify the surface morphology of the titanium alloys, the acid-etching process was carried out in two separate stages. In the first stage, samples were immersed in hydrofluoric acid (HF) for 15 s at room temperature with manual agitation to remove the hydrogen bubbles generated during the chemical reaction [17,30]. After the first acid-etching step, samples were cleaned in a sodium bicarbonate solution to neutralize the electrolyte and were then rinsed with purified water to eliminate residue. The second acid-etching step was carried out in a mixture of HCl and HF solution for 30 min, with pneumatic agitation to remove the hydrogen bubbles generated during the chemical reaction [21]. After the second acid-etching step, samples were washed in a sodium bicarbonate solution to neutralize the electrolyte and were then rinsed with purified water to eliminate residue. After being rinsed in water, samples were immersed in an ultrasonic cleaning tank with acid nitric solution for 10 min at 50 °C to eliminate the surface residue from the acid-etching process. Finally, samples were immersed in an ultrasonic tank with purified water for 20 min at 50 °C and were oven-dried at 100 °C for 1 h. Both acid-etching processes were carried out at room temperature. 

Metallographic preparation was carried out by acid etching with Kroll solution (3 mL HF, 6 mL HNO_3_, 100 mL H_2_O) to reveal its microstructure. A microstructural analysis was carried out to identify phases, morphology, homogeneity, and distribution by optical microscopy under a Nikon LV100 optical microscope (Nikon, Tokyo, Japan). The effect of double acid etching on the topography of the two titanium alloys was qualitatively analyzed by scanning electron microscopy (SEM, JEOL JSM 6300, Tokyo, Japan). The microchemical analysis of the surface was run with an Energy DispersiveSpectroscopy (EDS, Oxford Instruments Ltd., Oxford, UK). Surface roughness was analyzed by confocal microscopy (FRT CFM). Three measurements were taken for each specimen according to ISO 4287:1997. The 20X objective was employed, with 3 nm resolution on the “z” axis, and one of 1.23 µm on the “x” and “y” axes. The surface analysis area was 960 × 720 µm. Data analysis and 3D topography were performed with FRT Mark III^®^ (FRT GmbH, Bergisch Gladbach, Germany). The surface mean roughness (Sa), surface roughness depth (Sz), surface quadratic average roughness (Sq), and surface maximum roughness height (Smax) were calculated as the typical height parameters. Space descriptive parameters were calculated, including the value of the surface medium roughness of the peaks above a profile plane (Spk), the medium roughness value of the surface valleys below the profile plane (Svk), and the biggest difference between the surface maximum and average surface heights (Sp). This range of parameters was selected in order to include the relevant parameters capable of describing and explaining the surface topography of the studied titanium alloys.

## 3. Results

The Ti35Nb10Ta1.5Fe samples presented high densification—around 77% of the theoretical density after compaction, and about 94% after the sintering cycle. The mechanical characteristics of the studied Ti35Nb10Ta1.5 alloy obtained in this research are summarized in Table 1. The P/M Ti6Al4V and Ti CP Grade 2 properties are also reported in this table for comparison purposes. Studying the results of the bending test done on Ti35Nb10Ta1.5Fe revealed that higher values were obtained for ultimate tensile strength and lower elongation than for Ti CP Grade 2. An elevated amount of β stabilizing elements decreases diffusion during sintering; this effect translates into low grain size, which increases the ultimate strength values of the titanium alloy. Additionally, lack of diffusion results in an elevated residual porosity, which increases the stress concentration effect and decreases elongation. These facts explain that Ti alloys with lower values of porosity present higher values of elongation (Table 1). Another approach that can be employed to increase titanium alloys elongation values are stress relief heat treatments, which are normally carried out at 550–650 °C for around 1 h. Young’s modulus was one of the most crucial mechanical properties that controlled the load transfer between implant and bone. The increase in the beta stabilizer elements on Ti35Nb10Ta1.5Fe, compared with the Ti6Al4V and Ti CP alloys, also had a significant effect on lowering Young’s modulus. The studied β titanium alloy presented better mechanical properties than Ti CP, with a low Young’s modulus, which could minimize bone atrophy due to the stress shielding effect, and could increase implant durability.

**Figure 1:**
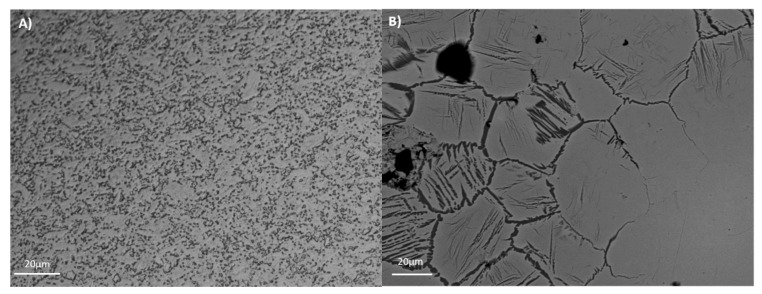
Microstructure of the studied titanium alloys. (**A**) Ti6Al4V ELI; (**B**) Ti35Nb10Ta1.5Fe.

The mechanical properties (e.g., tensile strength, wear, and fatigue) and corrosion resistance are directly related with alloy microstructure. The microstructures of the tested alloys are presented in Figure 1. For Ti35Nb10Ta1.5Fe, the white contrast areas are the β-phase plates (Figure 1B). The α-phase present among the β-phase regions gives rise to a dark contrast. The microstructure shows a heterogeneous phase composition, composed predominantly of a β equiaxial grain with a small amount of the remaining α phase due to the fabrication procedure. The α phase is present mainly on the grain boundaries, and also in an acicular form in those regions with lower contents of refractory elements. This effect is explained by the low chemical homogeneity due to poor diffusion. As the studied alloys presented more than a 20% retained β phase, at room temperature they were classified as β alloys [3]. Ti6Al4V ELI presented an α + β microstructure, composed of non-equiaxial grain due to cold working operation that reduces the grain size to sub-micrometer scale, with a fine dispersion of the alpha and beta phases that resulted from the processing done in the alpha plus beta field (Figure 1A).

The osteoblastic response depends on implant surface properties, where roughness and topographical features play an important role in the osteoconductive process [1,2,13,21,25]. Conventional surface treatments employed to modify titanium surfaces such as plasma spray or grit blasting are capable of modifying the surface roughness in ranges from 10 to 30 µm. Instead, the acid-etching is capable of modifying titanium surfaces in the microroughness range, between 1 to 10 µm. For this reason, the surface topography and surface roughness properties of acid-etched titanium alloys were studied. The surface images of the acid-etched samples revealed different topographical features between the studied β and α + β alloys. 

**Figure 2:**
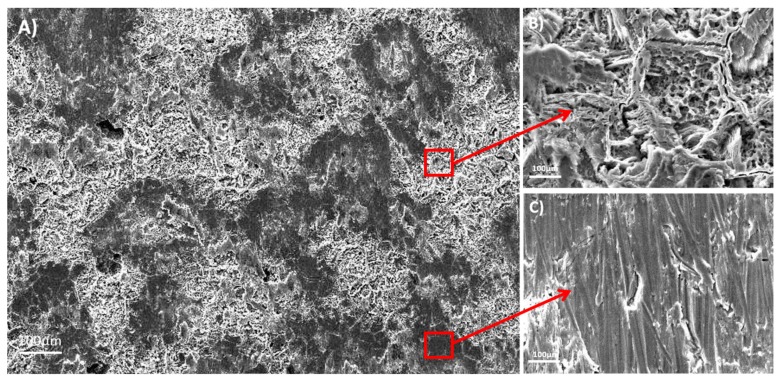
The SEM images for the acid-etched Ti35Nb10Ta1.5Fe. (**A**) General view; (**B**) The α phase region; (**C**) The β phase region.

The Ti35Nb10Ta1.5Fe alloy presented high microstructure heterogeneity due to the poor diffusion of the refractory elements during the sintering process, which translated into two differentiated topographies after the double acid-etching process. The α + β phase region presented high corrosion after the acid-etching treatment, along with deep craters and micropits (Figure 2B). S. Ban et al. obtained the same topography on the Ti CP etched in 48% H_2_SO_4_ at 60 °C [25]. The β phase region showed flat facets (Figure 2C) with minor irregularities (e.g., pits and stripes). These β regions displayed greater corrosion resistance with lower titanium dissolution that ended on the smooth surface. 

**Figure 3:**
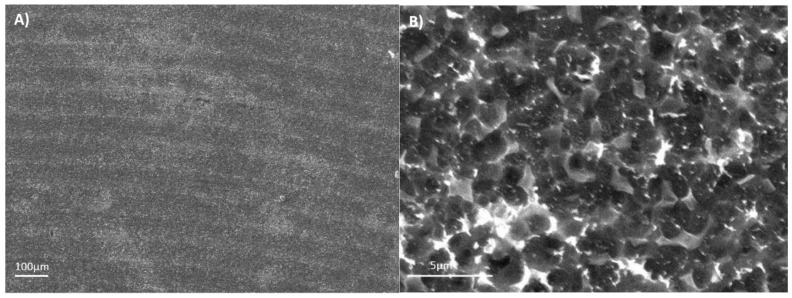
The SEM images for the acid-etched Ti6Al4V ELI: (**A**) 100×; (**B**) 1500×.

The Ti6Al4V ELI acid-etched surface had a heterogeneous surface topography with peaks and valleys of varied geometries, and the distance from peak to peak was around 1–3 µm. The differences in surface topography can be attributed to the non-equiaxed grains and phase distribution found on the studied alloy. Micropits were overlapped a macrorough textured area produced by the grooves from the machining process (Figure 3A).

The energy dispersive X-ray analysis results of the studied titanium alloys are summarized in Table 2. After performing surface acid treatment, the EDS analysis detected stable oxide formation on the Ti35Nb10Ta1.5Fe alloy surface, and the amount of detected oxygen was 7.5% in weight. The oxide layer formed spontaneously on the beta titanium alloy surface after the acid etching process is thick enough to be detected using EDS technique. This phenomenon, which was not detected for the Ti6Al4V ELI alloy, can be explained mainly by niobium and tantalum being highly reactive elements that form more stable and thicker oxide layers than titanium [19]. Several authors have demonstrated that a titanium dioxide layer enhances corrosion resistance, osteoblast differentiation, and bone growth [1,2,23].

**Figure 4:**
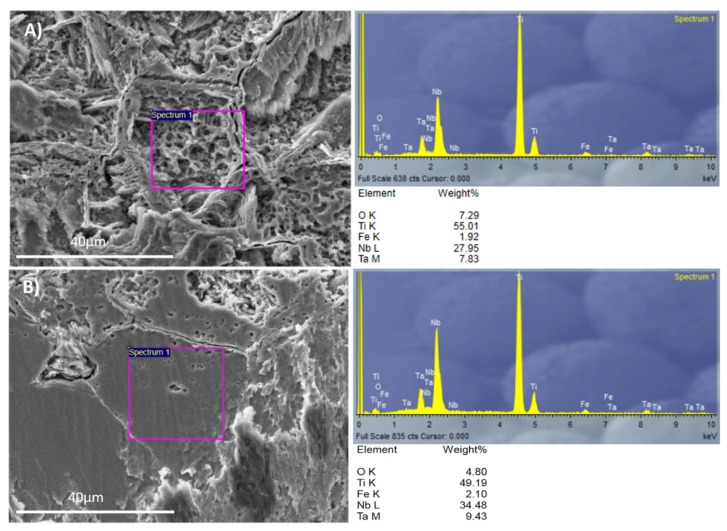
The EDS analysis for Ti35Nb10Ta1.5Fe: (**A**) The α + β phase region; (**B**) The β phase region.

The acid-etching process not only had different effects on the β alloy topography, but also changed the surface chemistry. The two regions identified by SEM for the Ti35Nb10Ta1.5Fe alloy were studied with EDS to evaluate the surface’s chemical composition. The EDS spectra and chemical composition are summarized in Figure 4. 

The contents of the beta stabilizer elements present in the region with high acid-etching corrosion and roughness values were lower than the region with facets. This allowed us to conclude that these areas were composed of the α + β phase, while the flat zones were composed of the β phase. The EDS results corroborated the metallographic analysis carried out by optical microscopy, as well as the results obtained by SEM. 

The values of the roughness parameters for the different Ti alloys studied herein are shown in Table 3. The Sa roughness parameter with double acid treatment gave an average roughness of between 0.06 and 0.20 µm. In the present work, the Sa values for Ti35Nb10Ta1.5Fe had two differentiated regions after the acid-etched process: the α + β region, with higher corrosion and values around 0.20 µm; the β region, with greater chemical resistance and Sa values that came closer to 0.06 µm. A major difference in the surface roughness depth (Sz), maximum roughness height (Smax), and average surface height (Sp) between the α + β phase and the β phase was detected in the Ti35Nb10Ta1.5Fe alloy. 

**Figure 5:**
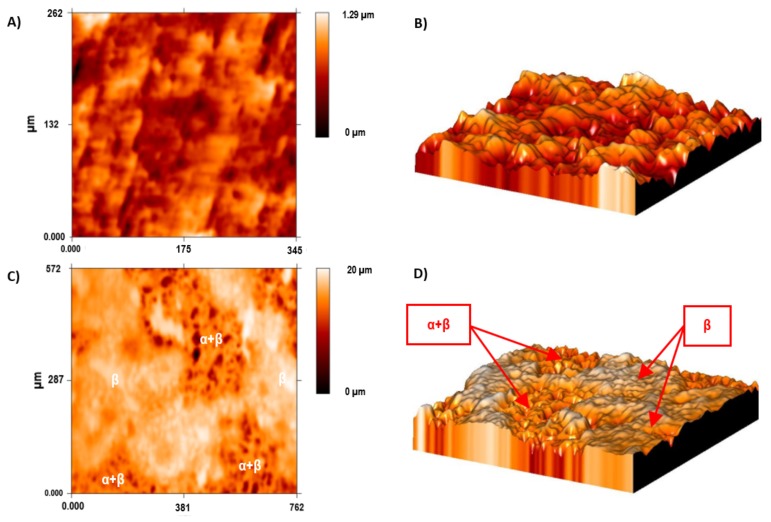
The surface roughness of the Ti35Nb10Ta1.5Fe alloy by confocal microscopy. (**A**) The 2D topographic roughness grid sample; (**B**) The 3D topographic roughness grid sample; (**C**) The 2D topographic roughness acid-etched sample; (**D**) The 3D topographic roughness acid-etched sample.

A bigger difference in the mean roughness values was observed in the α + β phase compared with the ground sample and the β phase of the acid-etched Ti35Nb10Ta1.5Fe samples. This phenomenon was also observed in the 2D and 3D profiles, obtained by employing the FRT Mark III^®^ software, where the more etched areas were plotted in dark red in Figure 5C and with deeper cavities in Figure 5D. 

**Figure 6:**
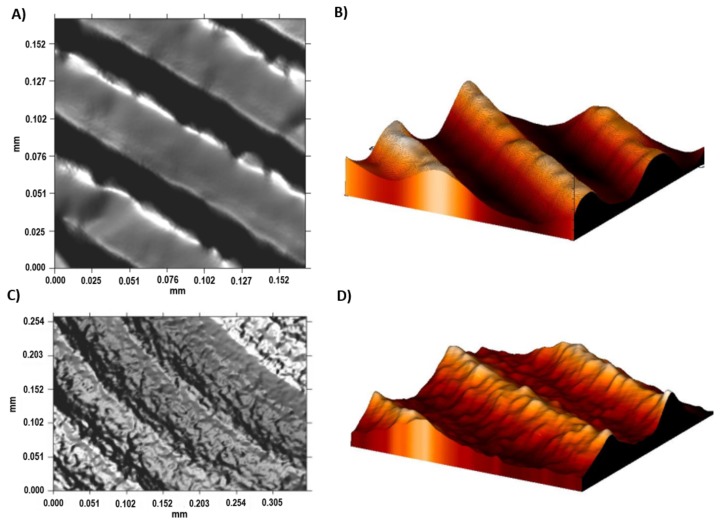
The surface roughness of the Ti6Al4V ELI alloy shown by confocal microscopy. (**A**) The 2D topographic roughness grid sample; (**B**) The 3D topographic roughness grid sample; (**C**) The 2D topographic roughness acid-etched sample; (**D**) The 3D topographic roughness acid-etched sample.

The Sa values of Ti6Al4V ELI roughness increased after the acid-etching process because the machined surfaces initially obtained an Sa value of 0.07 µm, which increased after acid etching to 0.09 µm. The difference between the topography of the machined and acid-etched surface of Ti6Al4V ELI are shown in Figure 6. The most relevant change was observed in the average surface heights (Sp) and the peak-to-valley roughness (Sz) for Ti6Al4V ELI after the double acid-etching process. After acid-etching treatment, the grooves from machining were covered with micropits of 13 nm in depth. This effect is observed in the 2D and 3D profiles shown in Figure 6C,D. Several studies have demonstrated that surface roughnesses within the 1–3 µm range are beneficial for the biochemical anchorage of oral implants [27]. 

## 4. Discussion

The double acid-etching process had different effects on the topography and surface chemistry of the two tested titanium alloys. The bigger difference in the roughness parameters was found between the α + β and the β regions of the acid-etched Ti35Nb10Ta1.5Fe samples. The acid etching on the Ti35Nb10Ta1.5Fe alloy produced micropits whose sizes ranged from 0.5 to 3 µm in the α + β regions (Figure 4A). Previous works have supported the hypothesis that this type of surface with microvoids allows better cell adhesion compared to implants without surface treatment [1,13,17]. Park et al. demonstrated that the double acid treatment on titanium surfaces increased the attachment of fibrin and osteogenic cells, and enhanced the osteoconductive process [33]. Nelson et al. reported that the removal torque of acid-etched implants is higher than machined implants, which means that the osseointegration mechanism is faster in acid-treated implants [34]. 

Apart from the differences in the topography obtained with both of the studied titanium alloys, the double acid-etched Ti35Nb10Ta1.5Fe samples presented a thicker and more stable oxide layer. The long-term implant performance is improved with a stable oxide layer, which lowers the ion release and the interactions with human cells [2,4,15,16,18]. Further studies are required to characterize the biocompatibility and long-term performance of beta titanium alloys alloyed with Fe, since this element is considered as cytotoxic when is employed as bulk metal.

The results of our present research are indicative of the fact that the double acid-etching process is a feasible treatment to obtain a nanosized topography on β titanium alloys. Further research into hydroxyapatite grit blasting and a strong acid-etching process (e.g., mixtures of H_2_SO_4_ and HCl) is required to increase the roughness profile of the β titanium implant surface. 

## 5. Conclusions

New β titanium alloys were synthesized using conventional powder metallurgy that contained a higher content of alloying elements and with a low Young’s modulus and adequate mechanical properties that render them suitable for use as biomedical implants. The elemental powder mixture yielded alloys offering a suitable microstructure and good mechanical properties despite their composition lacking homogeneity.

The studied alloys predominantly had a β-type microstructure. However, lack of diffusion produced mainly by Nb caused the presence of small α + β areas, which conferred alloys with sound microstructural heterogeneity. 

The double acid treatment not only changed the topography of the β alloy, but also altered the chemical composition of the surface. This surface treatment spontaneously created an oxide layer on the Ti35Nb10Ta1.5Fe alloy. This phenomenon is explained mainly by niobium and tantalum being highly reactive elements that form more stable and thicker oxide layers than the Ti6Al4V ELI alloy. Such a titanium dioxide layer enhances corrosion resistance, osteoblast differentiation, and bone growth.

The surface roughness of the Ti35Nb10Ta1.5Fe acid-etched sample correlated strongly with phase composition. A smaller difference in the mean roughness was observed between the ground sample and those found in the β phase region after the acid-etching process. 

The double etching process employed HF and a mixture of HF/HCl, and could provide two differentiated topographies on the Ti35Nb10Ta1.5Fe alloy required for various biological applications. 

## Figures and Tables

**Table 1:** Characterization of the studied titanium alloys.

Titanium Alloys	UTS (MPa)	ε (%)	E (GPa)	ρr (%)
Ti35Nb10Ta1.5Fe	669 ± 59	2.0 ± 0.1	78 ± 4	95 ± 1
Ti6Al4V [7]	960	12	110	99
Ti Cp Grade 2 [7]	414	15	103	96

Note: UTS = ultimate tensile strength, ε = elongation, E = Young’s modulus, ρr = relative density.

**Table 2:** The energy dispersive spectroscopy (EDS) results of the studied titanium alloys.

Elements (% Weight)	Acid-Etched Ti6Al4V	Acid-Etched Ti35Nb10Ta1.5Fe
Ti	92.9 ± 5.1	49.9 ± 4.8
V	3.9 ± 0.1	-
Al	4.9 ± 0.3	-
Nb	-	31.5 ± 3.3
Ta	-	8.9 ± 0.9
Fe	-	1.9 ± 0.2
O	-	7.5 ± 2.8

**Table 3:** The surface roughness parameters for the different studied alloys.

Roughness Parameters (µm)	Machined Ti6Al4V ELI	Acid-Etched Ti6Al4V ELI	Ground Ti35Nb10Ta1.5Fe	Acid-Etched Ti35Nb10Ta1.5Fe β Phase	Acid-Etched Ti35Nb10Ta1.5Fe α + β Phase
Sa	0.07 ± 0.003	0.09 ± 0.005	0.04 ± 0.010	0.06 ± 0.018	0.20 ± 0.033
Sq	0.08 ± 0.003	0.10 ± 0.005	0.05 ± 0.010	0.08 ± 0.020	0.25 ± 0.046
Sz (DIN)	0.34 ± 0.051	0.42 ± 0.046	0.32 ± 0.064	0.35 ± 0.112	1.22 ± 0.267
Smax	0.38 ± 0.010	0.48 ± 0.081	0.40 ± 0.038	0.44 ± 0.147	1.42 ± 0.307
Sp	0.20 ± 0.015	0.31 ± 0.083	0.16 ± 0.068	0.23 ± 0.079	0.53 ± 0.089
Spk	0.91 ± 0.025	0.13 ± 0.030	0.03 ± 0.007	0.06 ± 0.013	0.11 ± 0.089
Svk	0.03 ± 0.018	0.25 ± 0.012	0.06 ± 0.010	0.05 ± 0.028	0.34 ± 0.117
